# Striatal cholinergic interneuron regulation and circuit effects

**DOI:** 10.3389/fnsyn.2014.00022

**Published:** 2014-10-21

**Authors:** Sean Austin O. Lim, Un Jung Kang, Daniel S. McGehee

**Affiliations:** ^1^Committee on Neurobiology, University of ChicagoChicago, IL, USA; ^2^Department of Neurology, Columbia UniversityNew York, NY, USA; ^3^Department of Anesthesia and Critical Care, University of ChicagoChicago, IL, USA

**Keywords:** acetylcholine, cholinergic interneuron, Parkinson's disease, plasticity, striatum

## Abstract

The striatum plays a central role in motor control and motor learning. Appropriate responses to environmental stimuli, including pursuit of reward or avoidance of aversive experience all require functional striatal circuits. These pathways integrate synaptic inputs from limbic and cortical regions including sensory, motor and motivational information to ultimately connect intention to action. Although many neurotransmitters participate in striatal circuitry, one critically important player is acetylcholine (ACh). Relative to other brain areas, the striatum contains exceptionally high levels of ACh, the enzymes that catalyze its synthesis and breakdown, as well as both nicotinic and muscarinic receptor types that mediate its postsynaptic effects. The principal source of striatal ACh is the cholinergic interneuron (ChI), which comprises only about 1–2% of all striatal cells yet sends dense arbors of projections throughout the striatum. This review summarizes recent advances in our understanding of the factors affecting the excitability of these neurons through acute effects and long term changes in their synaptic inputs. In addition, we discuss the physiological effects of ACh in the striatum, and how changes in ACh levels may contribute to disease states during striatal dysfunction.

## Introduction

The striatum is a subcortical brain region crucial for integrating motivation and action (Da Cunha et al., 2012). Convergence of inputs from motor cortex, thalamus and limbic areas create associations between actions and outcomes that ultimately contribute to survival. The essential nature of the striatum is evidenced by the presence of homologs structures throughout vertebrate evolution over hundreds of millions of years (Stephenson-Jones et al., 2011). Pathological changes in the striatum and associated basal ganglia structures are implicated in a wide variety of neurological and psychiatric disorders that involve the combination of motivation and action, including drug addiction (Koob, 1992), binge-eating (Norgren et al., 2006), obsessive-compulsive disorder (Aouizerate et al., 2004), attention deficit and hyperactivity disorder (Chudasama and Robbins, 2006), Huntington's disease (Cepeda et al., 2007), Parkinson's disease (Albin et al., 1989; Ellens and Leventhal, 2013), and L-DOPA induced dyskinesia (Barroso-Chinea and Bezard, 2010). Because so many human pathologies involve striatal dysfunction, a better understanding of neurotransmission in this brain structure will provide insight into the etiology of these conditions, potentially leading to the development of new pharmacotherapies. One neurotransmitter that is highly enriched in the striatum and vitally important for normal function is acetylcholine (ACh). This review will focus on the regulation of striatal ACh release as well as the functional consequences of cholinergic neurotransmission.

Unlike brain structures that show ordered laminar organization such as the hippocampus or cortex, the striatum is a heterogeneous mix of different cell types. The vast majority of striatal neurons (~95%) are the GABA-ergic medium spiny neurons (MSNs), also referred to as spiny projection neurons, which are the principal output cell type. The MSNs that express dopamine (DA) D1 receptors project to and inhibit cells in the internal capsule of the globus pallidus as well as the substantia nigra pars reticulata. These projections are referred to as the direct pathway, or the GO pathway, and activation of this class of cells leads to enhanced locomotion. Another MSN population expresses dopamine D2 receptors, and these projections inhibit cells in the external capsule of the globus pallidus. This is the indirect, or the NO-GO pathway, and activation of this pathway decreases locomotion. Both pathways eventually influence thalamic control of motor cortex to affect motor function. Approximately 6% of MSNs in the dorsal striatum express both D1 and D2 receptors. These cells produce both GABA and glutamate, allowing them to potentially modulate the basal ganglia network bidirectionally (Perreault et al., 2012). The MSN network of basal ganglia connectivity has provided a model for understanding striatal involvement in motor control (Albin et al., 1989; DeLong, 1990; Kravitz et al., 2010). In addition to the MSNs, approximately 4% of striatal neurons are GABA-ergic interneurons. These locally projecting inhibitory cells consist of three types: parvalbumin-expressing fast spiking interneurons (FSIs), NPY/SOM/NOS-expressing persistent depolarization low-threshold spiking interneurons, and the less understood calretinin-expressing low-threshold calcium spike interneurons (Kawaguchi, 1993). Each of these GABA-interneuron cell types possesses a unique gene expression profile and distinct electrophysiological properties (Tepper et al., 2010).

The remaining cells are the large, aspiny cholinergic interneurons (ChIs) originally described by the anatomist Kölliker in the late 1800's. Although recent tract tracing and electron microscopy studies report cholinergic projections from the rostral pedunculopontine nucleus into the striatum (Dautan et al., 2014), it is generally accepted that ChIs are the main source of striatal ACh (Woolf and Butcher, 1981). ChIs comprise ~1% of striatal cells, yet they ramify extensively and send projections widely throughout the striatum: each ChI is estimated to produce on average 500,000 axonal varicosities (Bolam et al., 1984; Contant et al., 1996). Anatomically, ChIs are easily distinguished from the other striatal cell types due to their large diameter somata (>15 microns). In addition, ChIs display unique electrophysiological characteristics, which include tonic action potential firing at a rate of 3–10 Hz (Wilson et al., 1990), depolarized resting membrane potential (~ −60 mV) (Lee et al., 1998), high input resistance (~ 200 MΩ) (Calabresi et al., 1997), prominent hyperpolarization-activated cation current (I_h_) (Deng et al., 2007a), and broad action potential duration (Threlfell et al., 2012). The striatum has the highest levels of cholinergic markers in the brain, including ACh, choline acetyltransferase (ChAT), and acetylcholinesterase (AChE) (Macintosh, 1941; Hebb and Silver, 1961; Woolf et al., 1984). Such a high density of cholinergic markers underscores the importance of ACh neurotransmission in the striatum. Therefore, understanding striatal physiology requires careful consideration of the activity of ChIs and the consequences of changes in cholinergic signaling.

Classically, the striatum is subdivided according to synaptic connectivity. For example, the dorsal striatum receives DA-ergic input primarily from substantia nigra pars compacta (SNc) (Hattori et al., 1991) and sends projections to ventrolateral substantia nigra pars reticulata (SNr) and the globus pallidus. The ventral striatum receives the majority of DA from ventral tegmental area (VTA) projections, and in turn, sends inhibitory projections into the dorsomedial SNr (Maurin et al., 1999) and globus pallidus. In rodents, the dorsal and ventral striatum subserve different functions: The dorsal striatum is implicated in sensorimotor functions such as serial order learning (Yin, 2010), stimulus-response habit formation (Devan et al., 2011), and performance of learned instrumental tasks (Shiflett et al., 2010), whereas the ventral striatum is important for the reinforcement of appetitive behaviors including drugs of abuse (Robinson and Berridge, 2000) and healthy rewards, such as food intake (Kelley, 2004). Different functional roles are attributed to lateral vs. medial regions of the dorsal striatum. The dorsolateral region, or the “sensorimotor striatum,” receives strong motor and premotor cortical inputs, and is therefore particularly important for habit formation (Künzle, 1975; Haber et al., 2000). The dorsomedial striatum, referred to as the “associative striatum,” receives inputs from limbic regions as well as prefrontal cortex, and is involved in behavioral flexibility, reward-associated motor learning, and reaction time (Hauber and Schmidt, 1994; Ragozzino, 2003). Gradients of afferent connectivity most likely influence overall striatal output (Voorn et al., 2004), suggesting that these subdivisions are an oversimplification of striatal connectivity and function. Accepting that caveat, the distinctions in function of different striatal regions in the rodent brain provide a framework for ongoing investigations into the neural substrates of relevant behaviors, with an ultimate goal of understanding the functional role of analogous structures in the human brain.

ChIs are believed to be the analogs of tonically active neurons (TANs) identified by *in vivo* recordings in the putamen of primates. This correlation is based on similarities in ChAT immunoreactivity, electrophysiological, and morphological characteristics (Inokawa et al., 2010). Changes in TAN activity have been linked to motor and reinforcement learning. In classical sensorimotor Pavlovian conditioning, TANs pause activity within a second after presentation of the conditioned stimulus (CS), followed by a transient increase in activity before recovery to baseline firing. This stereotyped neural behavior was described as the “conditioned pause response” (Kimura et al., 1984; Aosaki et al., 1994). This CS-induced change in firing is not dependent on motor activity, as a similar firing profile was observed when the animal was trained to withhold movement after CS presentation in a NO-GO task (Apicella et al., 1991). TANs also pause in response to aversive-CS, but not to neutral stimuli (Ravel et al., 1999). The conditioned pause response is therefore believed to encode salience value to external stimuli. Thus, changes in TAN activity may contribute to associative learning, particularly the relationship between environmental cues and outcomes. The circuitry responsible for the pause response is debated. Some evidence implicates a dependence on SNc DA-ergic tone (Watanabe and Kimura, 1998; Reynolds et al., 2004; Straub et al., 2014), however others have observed a change in TAN firing even in response to aversive stimuli that do not increase DA-ergic firing (Mirenowicz and Schultz, 1996; Ravel et al., 1999). We know that ChIs respond to many neurotransmitters, and this stereotypical pause in activity could be mediated by a variety of inputs. Synchronous changes in afferent activity likely mediate the pause response among multiple ChIs, resulting in a coordinated change in striatal cholinergic tone. Understanding the connectivity and neurotransmission that influences these cells may thus provide insight into learning phenomena.

## Striatal cholinergic dysfunction in parkinson's disease and treatment

The necessity of proper striatal neurotransmission for normal motor function is dramatically and tragically evidenced by the deficits observed in Parkinson's disease (PD). The first medical characterization of PD was published in 1817 (Parkinson, 1817):

The first symptoms perceived are, a slight sense of weakness, with a proneness to trembling in some particular part; sometimes in the head, but most commonly in one of the hands and arms… After a few more months the patient is found to be less strict than usual in preserving an upright posture: this being most observable whilst walking.

Although this is the first formal description of the disease in Western literature, descriptions of the disease appeared in Eastern texts as old as 600 BC (Manyam, 1990; Zhang et al., 2006; Raudino, 2012; Ovallath and Deepa, 2013). Some ancient cultures used treatments derived from herbal preparations that contain anticholinergic compounds with similar pharmacology to some therapies prescribed today (Manyam and Sánchez-Ramos, 1999).

Today, we know that the bradykinesia, resting tremor, rigidity, and difficulty in initiating movement observed in PD arise from deficits in basal ganglia DA transmission. However, it is important to consider the balance between the actions of striatal ACh and DA in the etiology of PD. Previous beliefs held that the two neurotransmitters had opposing actions, which was supported by the partial relief of PD symptoms with administration of anticholinergic compounds. These therapies may restore the balance between the two neurotransmitter systems. Drugs with similar pharmacological properties are still in use, particularly for younger PD patients whose primary symptom is tremor (Hristova and Koller, 2000), but cognitive and autonomic side effects preclude their widespread use. While anticholinergic drugs can improve some symptoms of PD (Whyte et al., 1971; Cantello et al., 1986; Baba et al., 2012), it has also been reported that elevation of ACh by treating patients with acetylcholinesterase inhibitors improves motor symptoms of PD (Chung et al., 2010). Although somewhat contradictory to the anticholinergic drug effects, inhibiting ACh degradation might enhance DA transmission through nicotinic acetylcholine receptors (nAChRs) on DA terminals. Alternatively, this treatment could promote cholinergic receptor desensitization to mimic anticholinergic drug effects. It is important to note that similar cholinesterase treatments have seen no effect on motor symptoms of PD (Poewe et al., 2006). Together, these observations highlight the importance of cholinergic transmission in striatal function under healthy and Parkinsonian conditions.

The physiology of ChIs is dramatically altered in PD. In humans, mutations in leucine-rich repeat kinase 2 (LRRK2) or the gene that encodes α-synuclein are both associated with a higher likelihood to develop PD (Simón-Sánchez et al., 2009). These genes are expressed in many basal ganglia cell types and the mechanisms that link these mutations to PD are the subject of ongoing studies (Gasser, 2009). ChIs in both rodents and humans express high levels of LRRK2 (Higashi et al., 2007; West et al., 2014), and abnormal kinase activity may contribute to pathological changes in ChI physiology. α-synuclein inclusions in the somata of ChIs are observed only in late PD but not early PD (Mori et al., 2008), suggesting that Lewy body-related interference of ChI physiology may be observed late in the disease.

In the 6-OHDA lesion model of PD, microdialysis studies have observed that striatal ACh levels are elevated in the DA depleted rat striatum (DeBoer et al., 1993), indicating a dysregulation of ChI excitability. Additionally, functional downregulation of M4—Cav2 coupling results in decreased sensitivity to autoinhibitory cholinergic transmission (Ding et al., 2006). Given the therapeutic effects of anticholinergic compounds in PD mentioned above, and the physiological changes in ChIs seen in animal models of the disease, it is evident that ACh is important in PD.

The development of DA replacement therapy to relieve the symptoms of PD in the 1960's revolutionized our understanding of neurotransmission in the dorsal striatum (Goetz, 2011). Currently, the biochemical precursor to DA, levodopa (L-DOPA) is the most effective clinical treatment for the motor symptoms of PD. L-DOPA crosses the blood brain barrier where it is then converted into DA by aromatic amino acid decarboxylase, thus increasing striatal levels of DA. Although L-DOPA effectively reverses PD locomotor disability, long-term treatment has its shortcomings, including shortening of the therapeutic window and psychiatric or mood disturbances such as impulse control disorders (Lesser et al., 1979; Voon et al., 2009; Santangelo et al., 2013a,b). Another debilitating side effect is the onset of levodopa-induced dyskinesia (LID), which is characterized by dystonia or choreic movements of the limbs, hands, or face. These side effects are potentially more debilitating than PD itself. This condition is highly prevalent, with LID development seen in approximately 40% of patients after 5 years of treatment, rising to nearly 90% after 10 years (Ahlskog and Muenter, 2001; Fabbrini et al., 2007). Interestingly, the age of onset of PD is a strong determinant for the development of LID, with earlier onset patients experiencing a more rapid expression of LID symptoms (Kostic et al., 1991; Kumar et al., 2005).

Pathological changes in striatal ACh signaling are related to the expression of LID. The anticholinergic benzatropine decreased dyskinesia in L-DOPA treated human PD patients (Pourcher et al., 1989), however, there are also reports of increased dyskinesia with anticholinergic treatment (Birket-Smith, 1974; Hauser and Olanow, 1993; Linazasoro, 1994). In a study of dyskinetic monkeys, the mAChR antagonist atropine changed the nature of dyskinesia from dystonia to chorea (Gomez-Mancilla and Bédard, 1993). Although these reports show mixed effects, they do suggest that cholinergic signaling influences the expression of LID. While examining a mouse model of LID, Ding et al. (2011b) observed enhanced levels of phosphorylated extracellular signal-regulated kinase (pERK) specifically in striatal ChIs. Electrophysiological recordings of ChIs revealed higher baseline and dopamine-induced firing rates in LID animals relative to vehicle-treated littermates. The increased ChI excitability and the expression of LID associated behaviors were both inhibited by blockers of MEK/ERK signaling (Ding et al., 2011b). Extending those studies, selective ablation of striatal ChIs decreases LID expression in a unilateral lesion model of PD (Won et al., 2014). Both nicotinic and muscarinic receptors are believed to contribute to LID. Treatment with either nAChR antagonists or nicotine improves abnormal involuntary movements (AIMs) in rodents and primates, which suggests that both drugs are decreasing nAChR function either through receptor blockade or desensitization (Quik et al., 2007; Bordia et al., 2010; Zhang et al., 2013). Although antimuscarinic drugs have mixed effects in human LID patients as mentioned above, there are reports of decreased LID expression (Pourcher et al., 1989), and a recent study of the Pitx3 mouse model of LID, a muscarinic receptor antagonist decreased behavioral expression of dyskinesia (Ding et al., 2011b). These findings support the conclusion that cholinergic transmission is important for mediating some aspects of LID and that pharmacological modulation of this system may help treat this debilitating condition.

Changes in striatal cholinergic signaling have been observed in patients with other movement disorders and psychiatric illnesses. A partial list of these disorders and the nature of the changes in cholinergic activity can be found in Table 1. Considering the importance of striatal ChIs in both normal physiology and in disease, it is clear that this minority of cells plays a major role in the striatum. As such, understanding the nature of efferent and afferent synaptic connectivity of ChIs can provide important insights into striatal physiology.

**Table 1:** **Diseases associated with striatal cholinergic dysfunction**.

**Disorder**	**Nature of change**	**Species**	**Citations**
Parkinson's disease (PD)	Smokers are less likely to develop PD	Human	Morens et al., 1995; Allam et al., 2004; Quik et al., 2012
	↓ symptoms with anticholinergic drugs	Human (drug trial)	Katzenschlager et al., 2003; Lanska, 2010; Fox et al., 2011; Fernandez, 2012
	↓ AChE activity	Human (PET Scan)	Gilman et al., 2010
	↓ nAChR binding	Human (postmortem)	Rinne et al., 1991; Aubert et al., 1992; Court et al., 2000; Hellström-Lindahl and Court, 2000; Bohr et al., 2005; Gotti et al., 2006a
		Monkey (MPTP lesion)	Kulak et al., 2002; Quik and McIntosh, 2006
	↓ M1 binding	Human (PM)	Sirviö et al., 1989; Lange et al., 1993; Piggott et al., 2003
	Changes in CHRNB3 gene	Human (genotyping)	Bar-Shira et al., 2014
Huntington's disease	↓ in symptoms with AChE inhibitor	Rat (3-NP lesion)	Kumar and Kumar, 2009
	↓ ChAT activity	Human	Bird and Iversen, 1974; Enna et al., 1976a; Suzuki et al., 2001a
	↓ ChAT mRNA	Mouse (R6/1 model)	Smith et al., 2006
	↓ mAChR binding	Human (postmortem)	Hiley and Bird, 1974; Enna et al., 1976a,b
Alzheimer's disease	↓ cognitive deficits with AChE	Rat (ketamine induced behavior)	Zugno et al., 2013
	↓ AChE levels (in NAc)	Human (PM)	Hammond and Brimijoin, 1988
	↓ nAChR binding sites in putamen, but not in caudate	Human (postmortem)	Shimohama et al., 1985
	No change in nAChR binding	Human (postmortem)	Aubert et al., 1992; Gotti et al., 2006a
	↓ cognitive deficits with α7 or α4β 2 agonists	Human (drug trial)	Haydar and Dunlop, 2010
	↑ M1 binding	Human (postmortem)	Aubert et al., 1992
Schizophrenia	↑ likelihood to smoke	Human	Dalack et al., 1998; McEvoy and Allen, 2002
	↑ ChAT activity	Human (postmortem)	McGeer and McGeer, 1977
	↓ ChAT activity	Human (postmortem)	Bird et al., 1977
	↓ ChAT+ cells	Human (postmortem)	Holt et al., 1999
	↓ cognitive deficits with nicotine, α4β 2 agonist	Human (drug trial)	Radek et al., 2010
	↑ nAChR binding	Human (postmortem)	Court et al., 2000
	↓ nAChR binding	Human (postmortem)	Durany et al., 2000
	↓ mAChR binding	Human (SPECT scan)	Raedler et al., 2003
	↓ M1 levels	Human (postmortem)	Dean et al., 1996
	Changes in CHNRA7 gene	Human (postmortem, genotyping)	Leonard et al., 2002
Bipolar disorder	↓ β 2* nAChR binding	Human (PET scan)	Hannestad et al., 2013
	Changes in CHRNA7 gene	Human (genotyping)	Hong et al., 2004; Ancín et al., 2010
Tourette syndrome	↓ ChAT+ cells	Human (Postmortem)	Kataoka et al., 2010
	↓ tics with cholinesterase inhibitor	Mouse (DOI induced head tics)	Hayslett and Tizabi, 2003
		Human (drug trial)	Cubo et al., 2008
	↓ tics with nicotine	Mouse (DOI induced head tics)	Hayslett and Tizabi, 2003
		Human (drug trial)	Shytle et al., 1996; McEvoy and Allen, 2002
	↓ tics with nAChR antagonist	Mouse (DOI induced head tics)	Hayslett and Tizabi, 2003
		Human (drug trial)	Sanberg et al., 1998; Silver et al., 2000
	Alternative splicing in ACh related genes	Human (genotyping)	Tian et al., 2011
Attention Deficit Hyperactivity Disorder	No change in performance on attention tasks with nAChR agonist	Human (drug trial)	Jucaite et al., 2014
	↑ performance on attention tasks with nAChR agonist	Rat (MK801 induced attentional impairment)	Rezvani et al., 2012
		Human (drug trial)	Wilens and Decker, 2007; Bain et al., 2013; Potter et al., 2014
	Changes in choline transporter gene	Human (genotyping)	English et al., 2009
	Changes in CHRNA4 gene	Human (genotyping)	Todd et al., 2003; Lee et al., 2008; Guan et al., 2009; Wallis et al., 2009

## Afferent connections to ChIs

**Figure 1:**
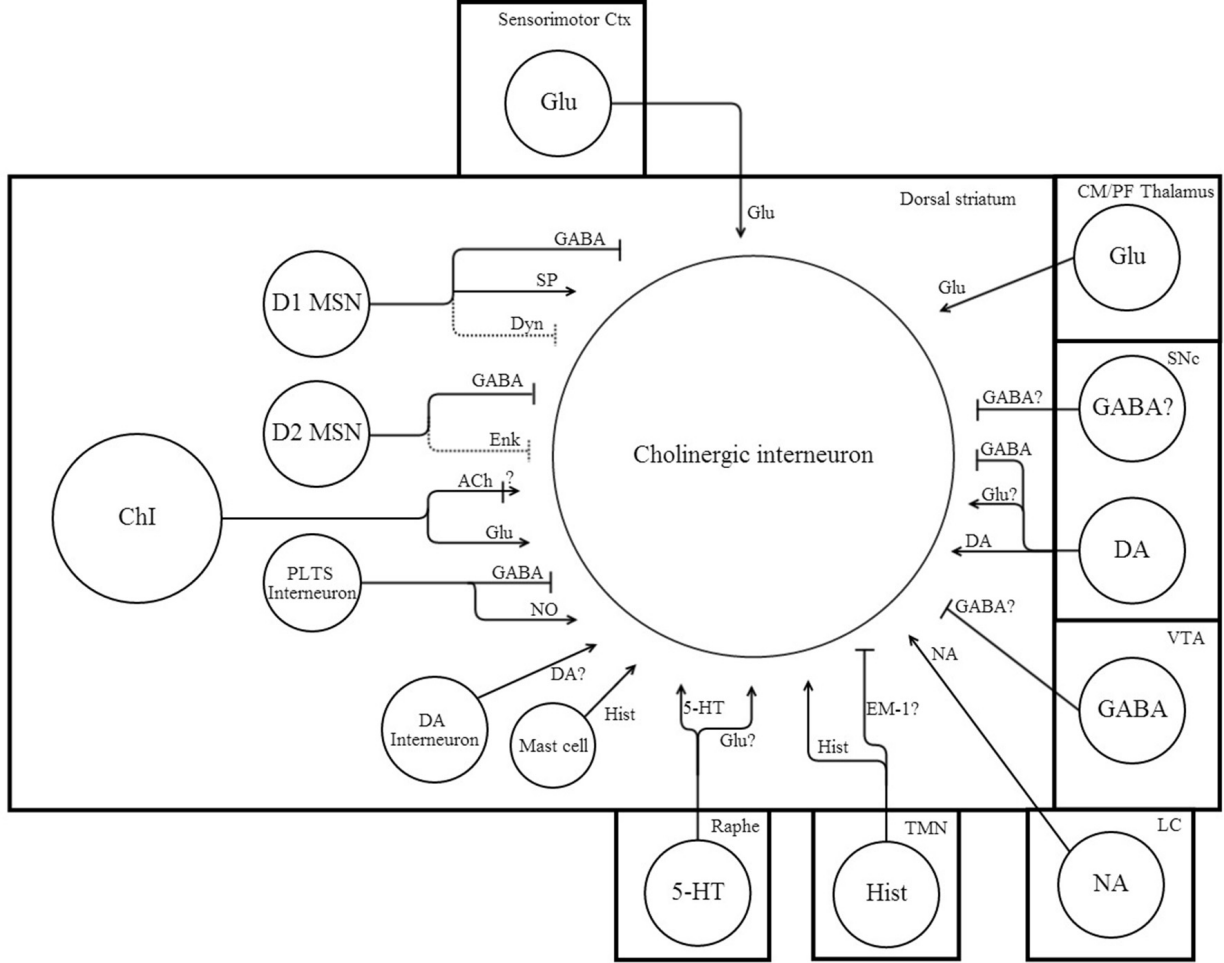
**Network connectivity of dorsal striatal cholinergic interneurons**. Simplified schematic illustrating some of the afferent synaptic inputs onto ChIs. Arrow heads indicate excitation, while perpendicular lines indicate inhibition. Dotted lines indicate a weak synaptic input. “?” denotes uncertain or untested inputs that are expected by either anatomical or physiological results. References that support afferent connections to ChIs can be found in the text and Table 2.

**Figure 2:**
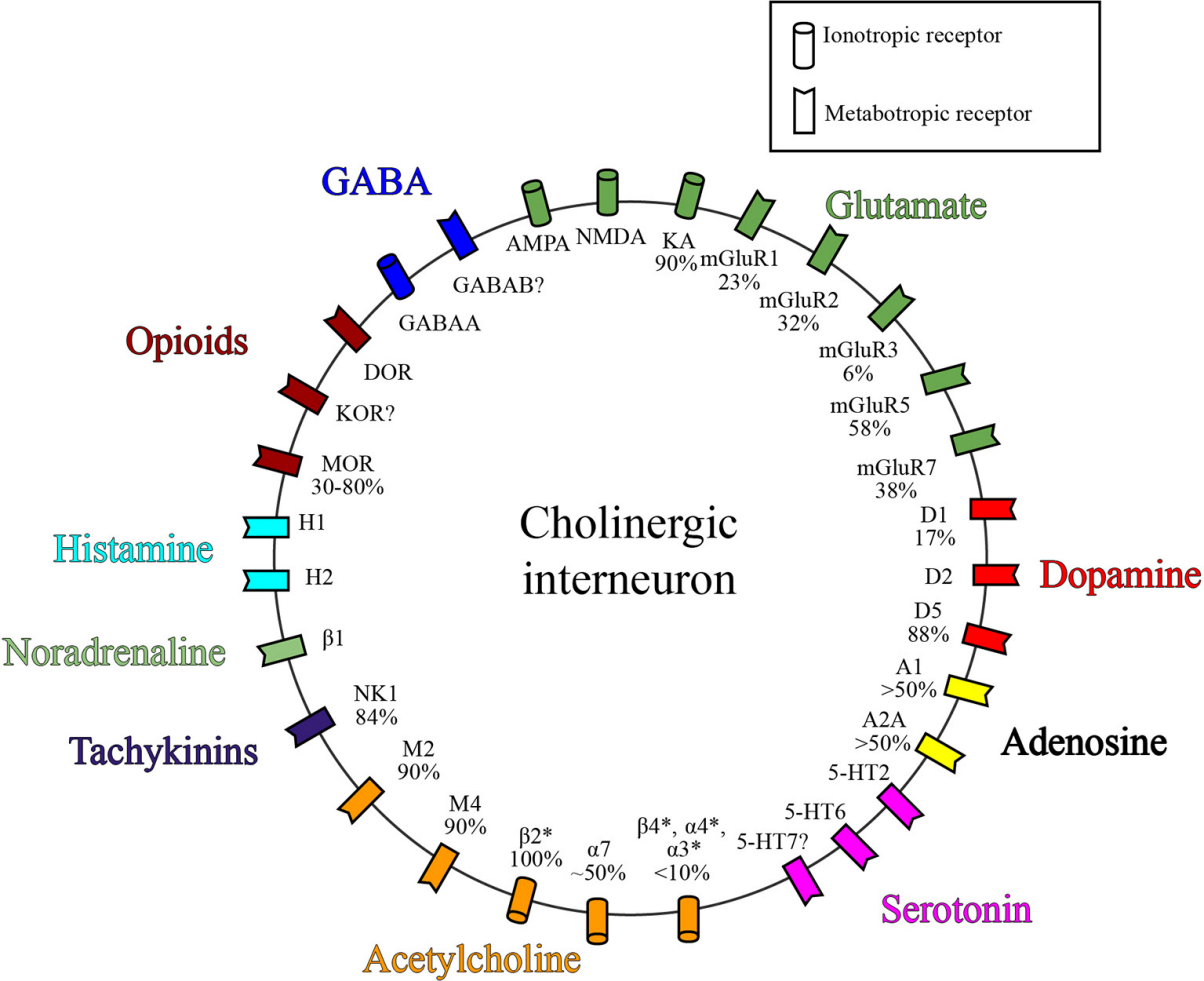
**Receptor expression on ChIs**. Simplified diagram depicting a subset of known receptor classes expressed on ChIs grouped by neurotransmitter. Values represent percent of ChIs that express the given receptor. References for receptor expression by ChIs can be found in the text and Table 2.

ChI excitability is affected by a remarkably large number of afferent input types and post-synaptic receptors. A simplified summary of this complex story is presented in Table 2 and Figures 1, 2. This section outlines the current state of our understanding of afferent control of ChIs.

**Table 2:** **Neurotransmitter systems and their effects on ChI activity**.

**Neurotransmitter**	**Source**	**Postsynaptic receptor targets**	**Effect on ChI**
GABA	MSN	GABA_A_	GABA_A_: Inhibition DeBoer and Westerink, 1994
	PLTS interneurons		
	SNc (?)		
Glutamate	Intralaminar thalamic nuclei	GluR1, 2, 4	AMPA, NMDA, Kainate: Excitation Calabresi et al., 1998b; Vorobjev et al., 2000; Cepeda et al., 2001
	Sensorimotor cortex	GluN1, 2D	mGluR1,5: Excitation Calabresi et al., 1999; Pisani et al., 2001; Berg et al., 2007
	SNc	Kainate	mGluR2: Inhibition Martella et al., 2009
	Raphe nucleus		mGluR7: No direct effect Bell et al., 2002
	ChI		
Dopamine	SNc	D1 (low levels)	Increased excitation Aosaki et al., 1998; Centonze et al., 2003; Ding et al., 2011b or
	DA-ergic interneurons	D2	Decreased excitation Deng et al., 2007a; Chuhma et al., 2014
		D5	
5-HT	Raphe nucleus	5-HT2	Increased excitation Blomeley and Bracci, 2005
		5-HT6	Excitation Bonsi et al., 2007
		5-HT7	Excitation Bonsi et al., 2007 or No effect Blomeley and Bracci, 2005
Histamine	TMN	H1	Depolarization and action potential firing Bell et al., 2000
	Mast cells	H2	
		H3	
Substance P	D1 MSNs	NK1	Depolarization, inward shift in holding current Aosaki and Kawaguchi, 1996
			Increased ACh release Arenas et al., 1991; Preston et al., 2000
Enkephalin	D2 MSNs	DOR	Decreased excitation Mulder et al., 1984
		KOR	Decreased excitation Schoffelmeer et al., 1997
			No effect on K+ induced ACh release Arenas et al., 1990; Jackisch et al., 1993
		MOR	Decreased excitation Ponterio et al., 2013
Dynorphin	D1 MSNs	KOR	Excitation at low concentrations of agonist Crain and Shen, 1996
			Inhibition at higher concentrations of agonist Gross et al., 1990
Noradrenaline	Locus coeruleus	β 1	Depolarization, increased action potential firing Pisani et al., 2003
Adenosine	Degradation of ATP	A_1_	Inhibition of ACh release Brown et al., 1990
		A_2A_	Increased ACh release Kurokawa et al., 1994
			No change in ACh release Jin and Fredholm, 1997
ATP	Synaptic release	P_2_X	No change in holding current Scheibler et al., 2004
		P_2_Y	
Nitric oxide	NOS+ PLTS interneurons		Depolarization Centonze et al., 2001

Summary of the neurotransmitter systems and other neuromodulators involved in the regulation of ChI activity. “?” denotes potential yet untested source of neurotransmitter release.

## GABA

ChIs receive a variety of GABA-ergic inputs, both local and extrastriatal in origin. GABA can inhibit cells by activating ionotropic GABA_A_ receptors, which increases Cl- conductance. Of the GABA_A_ receptor subunits, the α2, α4, β 2/3 subunits are most highly expressed in the striatum (Persohn et al., 1992), and of potential interest, the α3 subunit is expressed only in choline acetyltransferase positive (ChAT+) cells (Rodríguez-Pallares et al., 2000). Local stimulation produces IPSCs in ChIs, and these events are blocked by bicuculline, indicating that ChIs express functional GABA_A_ receptors (Sato et al., 2014). GABA also activates metabotropic GABA_B_ receptors, G-protein coupled receptors (GPCRs) which decrease cell excitation by coupling to the G_i/o_ protein and negatively regulating adenylyl cyclase (AC) (Bettler et al., 2004). Neither immunohistochemical examination of GABA_B_ expression in ChIs nor the electrophysiological effects of selective GABA_B_ activation on ChI excitability have been reported. *In vivo*, microdialysis experiments suggest that tonic activity at GABA_A_ receptors regulates ChI excitability, whereas the GABA_B_ receptors do not tonically inhibit ChI activity (DeBoer and Westerink, 1994).

Locally, GABA-ergic MSNs form synaptic connections with ChIs. Substance P-containing inputs are more prevalent than enkephalin-containing terminals, perhaps indicating that D1 MSNs have a more prominent influence over ChI activity compared to D2 MSNs (Martone et al., 1992). MSNs in the intact brain exist in one of two states of excitability, either an up or down state. In the up state, cells rest at a depolarized membrane potential, and are more likely to fire spontaneous action potentials compared to the relatively hyperpolarized down state (Wilson and Groves, 1981). The excitability state of MSNs will thus influence the inhibitory tone on ChIs. In the slice preparation MSNs are silent, and GABA released from these neurons will have minimal impact on ChIs. However, optogenetic activation of MSNs evoked a small amplitude IPSC in ~75% of ChIs, suggesting that the MSN-ChI connection is highly prevalent (Chuhma et al., 2011). Ultrastructural analysis of TAN connectivity in primates shows that approximately 24% of all synaptic contacts onto ChAT+ cells originate from MSN axon collaterals (Gonzales et al., 2013). In sum, GABA release from MSN onto ChIs is likely a major determinant of excitability of these cells *in vivo*.

ChIs also receive synaptic inputs from GABA-ergic interneurons. nAChR activation, presumably located on GABA-ergic interneurons, inhibits tonic firing of ChIs (De Rover et al., 2002; Sullivan et al., 2008). Not all GABA-ergic interneurons project to ChIs, however: PV positive FSIs project to MSNs and other interneurons, but do not inhibit ChIs (Szydlowski et al., 2013). Currently, NPY-expressing PLTS GABA-ergic interneurons are the best candidate for inhibition of ChIs, as ultrastructural analysis suggests synaptic connections with choline acetyltransferase-positive striatal neurons (Vuillet et al., 1992). However, there is no direct electrophysiological or functional evidence for this connection. It is unknown if calretinin positive interneurons form connections with ChIs.

Extrastriatal sources of GABA may also inhibit ChIs. Co-release of DA and GABA from nigrostriatal neurons onto MSNs occurs via a VMAT2-dependent vesicular mechanism (Tritsch et al., 2012). Cholinergic activation of nAChRs on those terminals was recently shown to enhance GABAergic inputs to MSNs (Nelson et al., 2014b). We know that DA projections form prominent synaptic contacts onto ChIs (Dimova et al., 1993; Li et al., 2002), and if some of these DA teminals co-release GABA, these inputs could profoundly affect tonic activity of ChIs *in vivo*. In addition, these projections may also have important implications in PD, as the loss of SNc projections could decrease this source of GABA-ergic tone onto ChIs, potentially contributing to increases in striatal ACh levels. Other non-dopaminergic projection neurons may contribute to GABA inhibition of ChIs. In addition to DA neurons, the VTA possesses GABA projection neurons that inhibit ventral striatum ChIs (Brown et al., 2012), but whether dorsal striatum ChIs receive GABA input from non-dopaminergic projection neurons in SNc or VTA is not known. Nigrostriatal non-dopaminergic projections have been observed (Gerfen et al., 1987; Rodríguez and González-Hernández, 1999), but the physiological effects of these presumed GABA projections on ChI activity have not been studied. These extrastriatal GABA projections may provide a means to inhibit ChIs that is independent of intrastriatal GABA sources.

## Glutamate – ionotropic receptors

Glutamatergic innervation of ChIs is predominantly extrastriatal (Künzle, 1975). Glutamate induces rapid depolarization through activation of postsynaptically expressed AMPA, NMDA, or kainate receptors. About half of all ChIs are immunopositive for GluR1, GluR2, and GluR4 subunits (Bernard et al., 1997; Deng et al., 2007b), despite the presence of mRNA of all 4 GluR subunits (Richardson et al., 2000). AMPA receptors expressed on ChIs show rapid deactivation, desensitization, and a relatively high permeability to Ca^2+^, and these properties differ from the AMPA receptors expressed by MSNs (Götz et al., 1997). mRNA for NR1 and NR2D are present at high levels in ChIs, while expression of NR2A mRNA is contested (Landwehrmeyer et al., 1995; Standaert et al., 1999; Richardson et al., 2000). 90% of ChIs are immunopositive for the kainate receptors GluR5/6/7 (Chen et al., 1996). In a slice preparation, bath application of an NMDAR positive allosteric modulator increases ChI firing rate (Feng et al., 2014), implying that glutamate tone in the slice preparation contributes to baseline ChI excitability. Considering the expression of these glutamate receptors on ChIs, it is not surprising that application of AMPA, NMDA, or kainate excites ChIs (Calabresi et al., 1998b; Vorobjev et al., 2000; Cepeda et al., 2001). Collectively, the expression of the three functional classes of glutamate receptors support the idea that glutamate is an important determinant of ChI excitability.

Electron microscopy has revealed that glutamate synapses comprise 13% of total synaptic connections onto ChIs (Gonzales et al., 2013). In the dorsolateral striatum, glutamate is released from cells located in the sensorimotor cortex and the centromedian/parafascicular nucleus of the thalamus, with the vast majority of excitatory projections being thalamic in origin (Berendse and Groenewegen, 1990; Lapper and Bolam, 1992; Thomas et al., 2000; Ding et al., 2010). Glutamatergic inputs, predominantly thalamostriatal projections are likely responsible for synchronous activation of ChIs, which has been suggested to coordinate DA release through activation of nAChRs on DA terminals (Ding et al., 2010; Threlfell et al., 2012). Another source of glutamate, corelease from SNc dopamine terminals, is a topic of debate—Optogenetic activation of SNc dopaminergic axons can produce a small amplitude EPSC in dorsal striatum MSNs (Tritsch et al., 2012). However, using a similar optogenetic approach, others report that glutamate and DA are only coreleased in the ventral, but not dorsal striatum (Stuber et al., 2010). Both of these studies were performed while recording from MSNs, and the electrophysiological significance of SNc-derived glutamate on ChI excitability has not yet been reported. Due to the close proximity between MSNs and ChIs, one would also expect an influence of this source of glutamate on ChIs (Dimova et al., 1993; Li et al., 2002). In this case, the loss of SNc projections in PD may impact the degree to which glutamate modifies ChI activity.

Serotonergic (5-HT) projection neurons from the dorsal raphe nucleus also express glutamate-like immunoreactivity. In culture, 5-HT cells form glutamatergic autapses, indicating that 5-HT projections may functionally co-release glutamate (Nicholas et al., 1992; Johnson and Yee, 1995). The extent to which changes in 5-HT transmission are associated with altered striatal glutamatergic signaling is unknown, but this may contribute to striatal dysfunction in mood disorders. SSRI treatment of depression will prolong the action of synaptically released 5-HT, and may lead to presynaptic inhibition through autoreceptors. This may alter local excitation via co-released glutamate to decrease striatal excitation.

Striatal ChIs also co-release glutamate with ACh (Higley et al., 2011). One isoform of the vesicular glutamate transporter, VGLUT3, is highly expressed in ChIs, and evidence suggests co-expression of this transporter with the vesicular ACh transporter on the same synaptic vesicles. These transporters act synergistically to optimize vesicular loading of ACh and glutamate (Nelson et al., 2014a). Vglut3 knock-out mice have a hypocholinergic striatum, presumably due to a decrease in loading of both glutamate and ACh into vesicles, and also due to less excitatory drive onto synaptically connected ChIs (Gras et al., 2008). The functional consequences of these non-thalamic/non-cortical sources of glutamatergic drive onto ChIs have not been studied in depth.

## Glutamate – metabotropic receptors

Glutamate also mediates long-term modulation of ChIs via metabotropic glutamate receptors (mGluRs). Excitatory group I mGluRs, which include mGluR1 and mGluR5, are highly expressed on ChIs (Tallaksen-Greene et al., 1998; Bell et al., 2002; Conn et al., 2005), and application of group I agonists induce excitation (Calabresi et al., 1999; Pisani et al., 2001; Berg et al., 2007). This excitation is mediated by a combination of cation currents through TrpC channels downstream of G_qα_, as well as inhibition of the chloride activated K^+^ channel Slo2.1 (Berg et al., 2007). The group II mGluRs, which consist of mGluR2 and mGluR3, decrease excitability by inhibiting AC through activation of G_i/oα_ (Diraddo et al., 2014). mGluR2, mRNA expression on ChIs (Testa et al., 1994; Bell et al., 2002) indicate that agonists of these receptors would theoretically decrease cell excitability. However, group II mGluRs are more involved in the modulation of synaptic inputs onto ChIs, as activation of these receptors results in no change in membrane potential, but decreases the amplitude of both excitatory and inhibitory synaptic inputs onto ChIs (Pisani et al., 2002; Martella et al., 2009). Of the group III mGluRs, only mGluR7 is expressed on ChIs, at a prevalence of 38% with no expression of mGluR4, 6, or 8. As with group II mGluRs, group III mGluRs decrease presynaptic release probability by inhibition of the AC pathway (Bell et al., 2002). Although rapid ChI excitation and inhibition are mediated by ionotropic receptors, it is important to consider that glutamate can have long term modulatory effects on ChI excitability via mGluR activation.

## Dopamine

In addition to GABA and glutamate, there are a number of other neurotransmitter systems that affect ChI activity. Striatal dopamine levels are the highest of any region in the brain and it is a principal determinant of striatal function. The predominant source of dopaminergic innervation of the dorsal striatum is A9 neurons—neurons which have cell bodies in the SNc and project broadly into the striatum, forming hundreds of thousands of synaptic connections per neuron (Kubota et al., 1987; Chang, 1988; Arbuthnott and Wickens, 2007; Moss and Bolam, 2008; Matsuda et al., 2009; Threlfell and Cragg, 2011). Although synaptic connections to MSNs are well documented, some reports demonstrate dopamine cells synapse onto ChAT positive cells (Hattori et al., 1976) while others report that dopamine modulates ChIs through volume transmission (Lehmann and Langer, 1983). These nigral dopaminergic neurons may exist in 4 different activity states. The tonically active state is independent of excitatory drive, as the neurons will fire at a rate of around 3 Hz *in vivo* or *ex vivo* in a slice preparation (Grace and Bunney, 1984; Hyland et al., 2002; Zhou et al., 2006; Ding et al., 2011a; Henny et al., 2012; Guatteo et al., 2013). They also can transition to burst activity with excitatory inputs increasing activity to around 20 Hz (Grace and Bunney, 1984; Hyland et al., 2002). In addition to these two active states, the cells may exist in one of two silent states, either hyperpolarization below action potential threshold, or depolarization block. Activity of these neurons is crucially important to normal striatal function (Gasser, 2009).

Postsynaptically, the majority of ChIs express D2 and D5 receptors with only about 20% of the neurons expressing low levels of D1 receptors (Dawson et al., 1988; Bergson et al., 1995; Yan et al., 1997). D2 receptors generally decrease neuronal exitability through activation of G_i/oα_, which also inhibits AC activity to decrease cAMP levels. D5 receptors are members of the D1 family that activate AC through G_s_ and generally increase excitability (Beaulieu and Gainetdinov, 2011). In the slice preparation, bath application of DA can strongly excite ChIs (Aosaki et al., 1998; Centonze et al., 2003; Ding et al., 2011b), however others have reported that DA inhibits ChIs by prolonging slow afterhyperpolarization duration (Deng et al., 2007a), and that optogenetic activation of DA terminals induces a pause in ChI firing (Chuhma et al., 2014). It was also reported that amphetamine-induced increases in striatal DA rhas no effect on ACh efflux *in vivo* (Abercrombie and DeBoer, 1997), implying that, under those conditions, elevated DA does not significantly affect cholinergic tone. Clearly, DA can affect ChI excitability, and the conditions under which DA either excites or inhibits these neurons will require further study.

In addition to this important extrastriatal source of DA, there also exists a small population of striatal DA interneurons in both primates and rodents (Dubach et al., 1987; Cossette et al., 2005; Ibáñez-Sandoval et al., 2010). Interestingly, the number of striatal TH+ cells increases following acute experimental dopamine depletion in both rodents and primates (Tashiro et al., 1989; Betarbet et al., 1997; Smith and Kieval, 2000; Jollivet et al., 2004). This is potentially a compensatory mechanism designed to counteract the loss of DA-ergic innervation from SNc. Strangely, the number of striatal TH+ cells is decreased in humans with PD (Huot et al., 2007), highlighting one difference between experimentally induced PD and the actual pathogenesis of the disease in humans. These DA interneurons form inhibitory GABA-ergic synapses with MSNs (Ibáñez-Sandoval et al., 2010). Whether or not these TH+ interneurons make DA-ergic and/or GABA-ergic synaptic contacts with ChIs is yet to be determined. Another interesting question to address would be whether or not these TH+ interneurons undergo changes in physiology that serve in a homeostatic role in the Parkinsonian striatum. Independent of changes in cell numbers outlined above, increased excitability following DA depletion may counteract low levels of striatal DA to help maintain striatal function. This small minority of striatal cells remains an interesting focus for future investigations.

## Acetylcholine

ChIs receive synaptic inputs from other ChIs. Both nAChRs and mAChRs are expressed at various levels on ChIs. With respect to nAChR expression, *in situ* hybridization has shown that all ChIs express mRNA for β 2 subunits, about half express α7 mRNA, while other subunit mRNAs are expressed at low levels (Azam et al., 2003). In support of the idea that ChIs express nAChRs, nicotine application to a slice preparation induces ACh release (Sandor et al., 1991). Interestingly, that effect was only seen in slices from animals that had undergone dopamine depletion with 6-OHDA treatment or in the presence of the D2 receptor antagonist sulpiride. These data suggest that resolving the nAChR-mediated ACh release requires elimination of D2 receptor mediated inhibition (Sandor et al., 1991).

The mAChR component of ACh modulation is through activation of the G_i/o_ coupled M2 and M4 receptors (Weiner et al., 1990; Smiley et al., 1999; Ding et al., 2006), thus acting as an autoinhibitory clamp to prevent excessive ACh release. No co-expression of M1 and ChAT is observed in the striatum (Dawson et al., 1990; Alcantara et al., 2001).

Synchronized activity in ChIs is observed following presentation of behaviorally salient stimuli (Apicella et al., 1997; Ravel et al., 1999). Although this synchronous firing has been linked to coordinated thalamostriatal inputs (Ding et al., 2010), ChI projections to other ChIs may also contribute to this synchrony through positive feedback control to coordinate strong increases in ACh. Whether or not nAChR mediated transmission contributes to ChI-ChI signaling has yet to be reported.

## Serotonin

Serotonin is a major determinant of ChI excitability. 5-HT-ergic projections originate from the raphe nucleus in the hind brain. These cells fire tonically at a rate of about 1–2 Hz (Innis and Aghajanian, 1987; Sprouse et al., 1989; Haj-Dahmane et al., 1991), releasing 5-HT into many brain areas including the striatum. 5-HT has an overall direct excitatory effect on ChIs, increasing action potential firing and membrane depolarization (Blomeley and Bracci, 2005; Bonsi et al., 2007). Activation of the G_q_-coupled 5-HT2 receptors increases excitability due to a decrease in the amplitude of both the slow and medium afterhyperpolarization (AHP) (Blomeley and Bracci, 2005). It has not been examined if 5-HT receptor classes 1A, 3, or 4 are expressed on ChIs, however selective agonists of these receptor classes do not induce changes in ChI excitability (Blomeley and Bracci, 2005). 5-HT6 activation excites ChIs (Bonsi et al., 2007). The contribution of the 5-HT7 receptor to excitation is debated, as Blomeley and Bracci (2005) reported no effect, while Bonsi et al. (2007) observed depolarization.

## Histamine

Histamine (HA) is a neurotransmitter that was first identified as a peripheral vasodilator with an effect on respiratory patterns and muscle tone (Dale and Laidlaw, 1910). Originally described in the brain in 1984, HA immunoreactive fibers were found to project widely throughout the brain including the striatum (Haas et al., 2008). HA-ergic cell bodies reside only in a small region of the posterior hypothalamus, the tuberomamillary nucleus (Panula et al., 1984; Blandina et al., 2012). Histamine is also produced by mast cells (Schwartz et al., 1986).

HA can act on 4 different types of G-protein coupled receptors, H1 through H4 (Parsons and Ganellin, 2006). These receptors are widely expressed, but only H1, H2, and H3 are highly expressed in the striatum, with H1 and H2 expressed on ChIs, and the autoreceptor H3 being expressed presynaptically on HA-ergic terminals. H1 and H2 are excitatory, coupling to G_q_ and G_s_ respectively, while the inhibitory H3 receptor is coupled with G_i_ (Timmerman, 1989; Haas et al., 2008). HA application has the net result of depolarization of ChIs, presumably acting through by H1 receptors (Bell et al., 2000). In the ventral striatum, ACh overflow is increased following H1 activation, while blockade of H2 increases ACh overflow, presumably through activation of H2 on GABA interneurons (Prast et al., 1999b). The same group found an increase in ACh overflow with concurrent H3 activation, an effect mediated by presynaptically expressed GABA interneurons (Prast et al., 1999a).

## Opioids

ChIs are also sensitive to opioidergic modulation. δ, κ, and μ opioid receptors (DOR, KOR, and MOR, respectively) are the three major classes of opioid receptors in mammals. These receptors inhibit cell activity through coupling with G_i/o_ proteins (Mansour et al., 1994; Tso and Wong, 2003), and are activated endogenously by a number of tightly regulated peptides. The endogenous opioid enkephalin activates DORs, and is produced by D2 expressing indirect pathway MSNs. mRNA for DORs is expressed in striatal ChIs (Le Moine et al., 1994), and activation of these receptors decreases ACh release (Mulder et al., 1984). The low number of synaptic connections between enkephalinergic cells and ChAT positive cells (Martone et al., 1992) suggests that enkephalin may only minimally inhibit ChI activity endogenously, but this does not preclude the possibility of volume transmission. Systematic investigation of endogenous DOR effects on ChI excitability has not been reported.

KOR is another major class of opioid receptors in the striatum. The endogenous opioid dynorphin is produced by D1 expressing direct pathway MSNs, and activates KORs. Like DORs, KORs are widely expressed in the striatum (Fallon and Leslie, 1986; Mansour et al., 1994). Compared to the DOR or MOR, the KOR receptor can be associated with G_i/o_ as well as G_s_. This bipolar effect of KOR activation is concentration dependent. At very low, subnanomolar concentrations of agonist, KOR preferably couples to G_s_ (Crain and Shen, 1996), but increasing the concentration results in the activation of signaling cascades downstream of G_i/o_ (Gross et al., 1990; Claye et al., 1996). Thus, depending on the level of striatal dynorphin, ChIs may either increase or decrease their excitability. KOR activation decreases ACh release in the striatum (Mulder et al., 1991; Schoffelmeer et al., 1997), however, different studies showed no effect of KOR activation on ACh release (Arenas et al., 1990; Jackisch et al., 1993). These apparently contradictory findings could result from the biphasic dose-dependent intracellular coupling of the KOR. It is also possible that the KOR effects on ACh release occur through indirect modulation, as there has not been a direct demonstration of co-localization of KOR with ChAT expression in striatum, nor is there direct electrophysiological evidence of KOR expression on ChIs.

The MOR is also coexpressed on ChAT positive striatal cells, however with tremendous diurnal variation, fluctuating from 30% coexpression in the daytime to a peak of 80% coexpression in the afternoon (Jabourian et al., 2005). Activation of MOR by exogenous DAMGO decreases ChI firing (Ponterio et al., 2013). MOR-inhibition of ACh release lowers the DA release probability in striatum by limiting activation of presynaptic nAChRs (Britt and McGehee, 2008). Endomorphin-1 (EM-1), an endogenous agonist at the MOR, shows only weak immunoreactivity in the striatum. EM-1 may be co-released by histaminergic neurons, as the EM-1 immunoreactivity signal is very prominent in the posterior hypothalamus (Martin-Schild et al., 1999). In addition, some endogenous agonists have overlapping affinity for different opioid receptor classes, such as Leu-enkephalin, which activates both DORs, and MORs at physiological concentrations (Jabourian et al., 2005).

## Tachykinins

Tachykinins are another class of neuropeptides expressed in the striatum. In addition to producing GABA and dynorphin, D1-expressing MSNs also express the tachykinin Substance P Terminals that contain Substance P. form synaptic connections with ChIs. Substance P. is a potent activator of NK1 receptors, which are expressed by ChIs (Bolam et al., 1986; Richardson et al., 2000). Activation of NK1 results in excitation (Aosaki and Kawaguchi, 1996) and increased ACh release (Arenas et al., 1991; Preston et al., 2000).

## Efferent connections of ChIs

Even though the ChIs make up a small fraction of cells in the striatum, they possess a large synaptic arbor and thus send ACh projections broadly throughout the striatum. As such, changes in ChI physiology influence a multitude of postsynaptic targets by activation of nicotinic and muscarinic ACh receptors. This section addresses the effects of ACh neurotransmission.

## Nicotinic acetylcholine receptors

Nicotinic acetylcholine receptors (nAChRs) are ligand-gated, pentameric ion channels that are activated by endogenous ACh, exogenous nicotine, or other ligands. nAChRs can be expressed both pre and postsynaptically, where they induce depolarization and increase excitability. Presynaptic nAChRs enhance release of several different neurotransmitter types (MacDermott et al., 1999). The subunits that are assembled into neuronal nAChRs include α2-α10 and β 2-β 4 (Patrick et al., 1993; McGehee and Role, 1995; Dani, 2001). They can be composed of homomeric or heteromeric subunit combinations, which determine characteristic pharmacological and biophysical properties of the receptor (Fenster et al., 1997; Gotti et al., 2006b). In the striatum, the most common nAChR subunits are the α4, α6, α7, β 2, and β 3, although other subunits are present at lower levels (Quik et al., 2007). Generally, nAChR activation induces rapid depolarization, but Ca^2+^ entry, particularly through homomeric α7 nAChRs can lead to rapid changes in neurotransmitter release or long term changes in cellular function through activation of Ca^2+^ dependent intracellular cascades, such as altered transcription through pCREB activation (Mulle et al., 1992; Chang and Berg, 2001; Hu et al., 2002; Wu et al., 2009; Del Barrio et al., 2011).

## Muscarinic acetylcholine receptors

In comparison to the rapid, excitatory effect of nAChR activation, mAChR activation serves a more long-term modulatory role. Activation of mAChRs can either increase or decrease cell excitability. A total of 5 subtypes of mAChRs have been isolated and cloned, but they are generally divided into 2 classes based on differences in their intracellular signaling cascades. The excitatory mAChRs, consisting of M1, M3, and M5, couple to G_q/11_ and induce activation of the phospholipase C pathway (Lin et al., 2004). The inhibitory receptors, M2, and M4, couple to G_i/o_ proteins and decrease activity of adenylyl cyclase (Wess, 1996). All 5 mAChRs are expressed in the striatum (Yan et al., 2001), however M1 and M4 are more heavily expressed than other isoforms, with a small presence of M2 and very low levels of M3 and M5 expression (Yasuda et al., 1993). Muscarinic receptors are not limited to somatic expression, as terminal expression of mAChRs serves to modulate neurotransmitter release probability. Expression of M2 receptors on ChI terminals serves an autoinhibitory role (Hersch et al., 1994). Additionally, neurotransmitter release at incoming afferents can be sensitive to mAChR modulation, as these mAChRs can receive synaptic inputs from ChIs. Because mAChR activation can either increase or decrease cell excitability, the net effect of ACh release depends on the patterns of postsynaptic mAChR expression. For each major striatal postsynaptic target, both the nicotinic and muscarinic effects on neuronal excitability will be addressed.

## Medium spiny neurons

GABA-ergic MSN projection neurons are the sole output of the striatum. Direct activation of AChRs on MSNs therefore represent a direct effect of ACh on striatal output. MSNs are generally believed to lack nAChRs (Matsubayashi et al., 2001; Luo et al., 2013), although Liu et al. (2007) reports that direct activation of nAChRs on MSNs by nicotine induces depolarization. This direct nAChR modulation of MSN activity has not been explored in depth, as the evidence that MSNs express nAChRs is quite limited. Interestingly, lesion studies indicate that only about 20% of α4β 2 nAChRs are expressed on DA terminals (Quik and Wonnacott, 2011). Thus, the contribution of these receptors to striatal circuitry likey involves expression on GABAergic interneurons and presynaptic projections from a range of cell types. Resolving the complete physiological role of striatal α4β 2 receptors is a topic of ongoing investigations.

The majority of studies of ACh-mediated modulation of MSNs focuses on mAChR activation. Bath application of the mAChR agonist carbachol increases MSN excitation in the absence of synaptic input, both in a slice preparation and in dissociated cell culture (Hsu et al., 1996; Galarraga et al., 1999). Two mechanisms have been proposed to explain this excitation. One involves an M1 mediated decrease in the inhibitory KCNQ potassium (Kv7) current (Shen et al., 2005), while the other an M1 mediated inhibition of Ca^2+^ entry through N and P/Q type channels, which in turn decreases the duration of the AHP (Pérez-Garci et al., 2003; Perez-Rosello et al., 2005). Neither of these studies differentiates between the direct and indirect pathway MSNs, and the mechanisms could differ between these cell types. Inhibitory M4 receptors are expressed on a subpopulation of MSNs (Bernard et al., 1992), and functional electrophysiological evidence suggests that M4 decreases Ca^2+^ influx to decrease excitability (Howe and Surmeier, 1995). Direct pathway MSNs express both M1 and M4, while indirect pathway MSNs express M1. Less than half of indirect pathway MSNs express M4 (Bernard et al., 1992; Yan et al., 2001). Both classes of MSNs would be excited with M1 activation, but the differential expression pattern of the inhibitory M4 could mean that ACh influences the two classes of MSNs in opposing directions.

## GABA-ergic interneurons

In addition to directly acting on MSNs, ACh can also modify striatal output through receptors on GABA interneurons that project to MSNs. Optogenetic activation of cholinergic cells produced IPSCs and IPSPs in MSNs that were inhibited by nAChR blockade. This microcircuit is believed to be a disynaptic connection, consisting of nAChR-expressing GABA interneurons that are activated by ACh, which then release GABA onto MSNs (English et al., 2011). The GABA interneurons that contribute to this inhibition of MSNs are likely the parvalbumin-expressing FSIs (Chang and Kita, 1992) and/or the NPY-expressing PLTS interneurons (English et al., 2011). However, English and coworkers did not observe an involvement of FSIs in the ChI-MSN interaction. Consistent with nAChR activation leading to elevated striatal GABA, inhibition of α7 receptors resulted in a decrease in striatal GABA in awake behaving animals (Beggiato et al., 2013), while activation of α7 nAChRs increases GABA levels (Campos et al., 2010).

Muscarinic receptor activation of GABA interneurons that project to MSNs can influence striatal output. Subcellular localization of the M2 receptor has been demonstrated in the NPY+ PLTS interneurons (Bernard et al., 1998). Consistent with this result, ACh decreases striatal GABA release (Marchi et al., 1990). More specifically, this is mediated by an inhibitory mAChR, as muscarine decreases GABA release onto MSNs (Sugita et al., 1991). Thus far, there are no reports of M1 receptor expression on GABA interneurons, but it is possible that M1-mediated enhancement of GABA output from one of the other interneuron subtypes neurons could contribute to striatal circuitry.

## Glutamatergic terminals

Glutamatergic inputs into the dorsal striatum originate primarily from the intralaminar nuclei of the thalamus and from the sensorimotor cortex, with a small amount of glutamate co-released from other terminals as well (Higley et al., 2011). nAChR expression on glutamatergic terminals provides a mechanism for cholinergic enhancement of excitatory drive onto MSNs. Increased glutamate release through activation of presynaptic nAChRs has been observed in brain regions such as the hippocampus, medial habenula, olfactory bulb and human neocortex (McGehee et al., 1995; Gray et al., 1996; Fisher and Dani, 2000; Girod et al., 2000; Marchi et al., 2002). Glutamate release probability is also modulated by nAChRs in the striatum (Kaiser and Wonnacott, 2000). *In vivo* microdialysis studies demonstrate that α7 nAChR activation in striatum increases glutamate release (Campos et al., 2010). α7 nAChR antagonism decreases glutamate release (Carpenedo et al., 2001), indicating that baseline ACh levels contribute to glutamatergic tone. Because the homomeric α7 subtype is highly Ca^2+^ permeable compared to other nAChRs stoichiometries, Ca^2+^ entry through these receptors may lead directly to enhanced neurotransmitter release (Gray et al., 1996). Activation of the α4β 2^*^ subtype also increases glutamate release onto MSNs (Xiao et al., 2009). As glutamatergic inputs originate from various neuronal types and brain regions, differential expression of nAChR stoichiometries may allow ChIs to amplify glutamate inputs differentially.

In contrast, activation of mAChRs negatively modulates striatal glutamate release. In field potential recordings, a mAChR agonist suppressed corticostriatal glutamatergic transmission (Malenka and Kocsis, 1988). Increasing mAChR signaling either by increasing ChI firing rates, or exogenous agonist application decreases excitatory drive onto MSNs (Calabresi et al., 1998a; Pakhotin and Bracci, 2007; Pancani et al., 2014). Muscarinic modulation of glutamatergic terminals occurs through M2 or M4 receptor activation, as mRNA and protein levels for both mAChRs are observed at high levels in striatal somata as well as terminals (Levey et al., 1991; Hersch et al., 1994). M2 or, interestingly enough, M3 activation results in paired pulse facilitation, indicating that mAChR activation decreases glutamate release probability (Hernández-Echeagaray et al., 1998; Ding et al., 2010). The change in release probability is observed in both corticostriatal afferents and thalamostriatal afferents, and when recording from both direct and indirect MSNs, indicating that regardless of the origin of the terminal or the post-synaptic target, release probability at glutamatergic terminals is decreased with mAChR activation (Ding et al., 2010). In agreement with these observations, intrastriatal injections of an M2-selective antagonist increases glutamate overflow (Smolders et al., 1997), providing evidence that tonic levels of ACh contribute to striatal glutamate tone. Additionally, glutamate release is downregulated via mAChR activation (Dodt and Misgeld, 1986). ChIs are thus in a position to regulate excitatory inputs to MSN, both rapidly by acting on nAChRs, and more slowly and persistently via mAChR activation.

## Dopaminergic terminals

ACh profoundly modulates DA release in the striatum. In a slice preparation, optogenetic activation of ChIs increases evoked DA release. The quantity of DA released is dependent on frequency of stimulation, and requires synchronous ChI cell activation as well as activation of β 2-containing nAChRs expressed on dopaminergic terminals (Cachope et al., 2012; Threlfell et al., 2012). Although the physiological conditions that coordinate synchronous firing of large numbers of ChIs are unknown, cross-talk between ChIs may facilitate simultaneous firing of these neurons to enhance DA release.

Dopaminergic terminals express α4 and β 2 subunits at high levels, along with α5, α6, α7, and β 3 subunits at variable levels (Le Novère et al., 1996; Sharples et al., 2000; Jones et al., 2001; Klink et al., 2001; Quik et al., 2003; Grady et al., 2007; Keath et al., 2007). Nicotinic agonists increase the efflux of DA in striatal tissue, as measured by microdialysis (Puttfarcken et al., 2000; Campos et al., 2010), and as expected, nAChR antagonists decrease DA efflux by interfering with the effects of local ACh activation of presynaptic nAChRs on DA terminals (Wonnacott et al., 2000; Grady et al., 2007). There is evidence that enhancement of DA release by exogenous activation of nAChRs requires glutamatergic signaling (Garcia-Munoz et al., 1996; Wonnacott et al., 2000), but enhancement of DA release by coordinated ACh release from ChIs is not dependent upon glutamate transmission (Threlfell et al., 2012). Additionally, striatal dopaminergic terminals also corelease GABA. In a recent study, optogenetic activation of ChIs produces a GABAA receptor mediated synaptic response in MSNs. Pharmacological blockade of α4 nAChRs inhibits this GABA current, suggesting that striatal nAChRs regulate GABA levels via modulation of release probability from DA terminals (Nelson et al., 2014b).

The expression of nAChRs on DA terminals not only enhances DA transmission, chronic agonist exposure, such as that achieved in tobacco users can shift DA release probability to suppress release during low frequency activity, but maintain or enhance release during burst firing (Zhou et al., 2001; Rice and Cragg, 2004; Zhang and Sulzer, 2004). These observations were obtained using high resolution fast-scan cylclic voltammetry to assess extracellular DA levels, and they suggest that nicotine may enhance the impact of high frequency DA neuron activity to effectively increase the salience of environmental stimuli. Recent *in vivo* investigations suggest that a chronic nicotine exposure model, which mimics the daily pattern of nicotine exposure by smokers (2 weeks via drinking water), results in a down-regulation of electrically stimulated DA release due to persistent desensitization of nAChRs on DA terminals (Koranda et al., 2014). This observation extends the results seen in other preparations including brain slices and non-human primates (Perez et al., 2012, 2013; Exley et al., 2013). Together, these findings have led to the intriguing speculation that the reported protective effects of smoking against PD may result from an adaptation in striatal circuitry to lower DA levels, thus delaying the onset of symptoms (Koranda et al., 2014).

Exogenous nicotine affects striatal dopamine in interesting and sometime counterintuitive ways, as the interplay between nAChR activation and desensitization can lead to contradictory effects. In contrast, endogenous ACh from ChIs is rapidly degraded by acetylcholinesterase, which is expressed at remarkably high levels in striatum. As alluded to above, synchronous ChI activation can have profound effects on DA release in striatum, through the coordinated activation of presynaptic nAChRs on DA terminals (Threlfell et al., 2012). This study from the Cragg laboratory used optogenetic stimulation to coordinate ChI activity selectively to demonstrate this phenomenon. This is relevant to endogenous activation of ChIs, as synchronous stimulation of these neurons has been reported through coordination of thalamostriatal inputs in response to salient environmental stimuli (Ding et al., 2010; Threlfell et al., 2012).

While mAChRs are also involved in modulation of DA release, the identity of mAChRs expressed by SNc DA cells is unclear, as some observe M2, M4, and M5 (Vilaró et al., 1990; Levey et al., 1991), while others report expression of only M5 receptors (Weiner et al., 1990). Agreement on M5 receptor expression suggests that dopaminergic terminals in the striatum express this mAChR subtype. Electrophysiological evidence supports this, as M5 KO mice show reduced oxotremorine enhancement of potassium-stimulated dopamine release (Zhang et al., 2002). M2 receptors are also involved in tonic DA release, as intrastriatal administration of an M2 antagonist dramatically increases DA levels in freely moving rats (Smolders et al., 1997). Non-selective activation of striatal mAChRs with oxotremorine increases DA release (Lehmann and Langer, 1982; Threlfell et al., 2010), suggesting that M5 activation plays a stronger role in the modulation of DA terminals.

## Plasticity of the excitatory inputs to cholinergic interneurons

Activity-dependent modification of synaptic connections is believed to be an important cellular substrate for learning and memory. As the dorsal striatum is believed to be an important site of action for habit formation and motor learning, it is likely that synaptic plasticity contributes to that learning. Considerable effort has focused on understanding the plasticity of the excitatory inputs to MSNs (Calabresi et al., 1996; Mahon et al., 2004; Surmeier et al., 2007), however, LTP/LTD in ChIs has not been explored extensively. Recording in tissue slices from dorsal striatum, Suzuki et al. (2001b) demonstrated LTP in ChIs following a 1 s, 100 Hz train stimulation of the corpus callosum. This LTP was dependent on Ca^2+^ entry, as intrapipette BAPTA blocked LTP induction. The source of Ca^2+^ entry in these studies was not from NMDA receptors, as NMDA blockers had no effect on LTP induction. They also found this LTP to be D5 receptor dependent, as pharmacological blockade of D1/D5 receptors prevents the long term maintenance of enhanced synaptic strength, whereas D2 receptor blockade did not affect LTP induction (Suzuki et al., 2001b). Bonsi and co-workers observed a similar LTP (using three 1 s, 100 Hz trains), however they attribute LTP at these synapses to calcium entry via L-type HVA channels, and not to Ca^2+^ permeable AMPA receptors or NMDA receptors (Bonsi et al., 2004). Using the same HFS stimulation paradigm as Bonsi et al, Picconi and coworkers demonstrate that plasticity of ChIs is not observed in the R6/2 mouse model of Huntington's disease (Picconi et al., 2006). It is not clear why a Huntington's disease model should lack plasticity at these synapses, which highlights the need for better understanding of the underlying mechanisms and functional significance of synaptic plasticity of the inputs to striatal ChIs.

Spike-timing dependent plasticity (STDP) is another experimental paradigm used to induce plasticity. In accordance with Hebbian theory, changing the time between presynaptic activation and postsynaptic depolarization can elicit either a strengthening, weakening, or no change in synaptic strength. First discovered in the cortex, examples of STDP have been observed in other parts of the nervous system including the hippocampus, striatum neuromuscular junction, and cerebellum (Linden et al., 1991; Markram et al., 1997; Wan and Poo, 1999; Nishiyama et al., 2000; Plotkin et al., 2013). To date, only one group has reported STDP in ChIs. Fino and colleagues observed bidirectional plasticity in a majority of ChIs—Post-pre stimulation elicited an LTP in some cells and LTD in others, while pre-post stimulation results in only LTD. Using pharmacology, they found that both forms of plasticity depend upon mGluR activation (Fino et al., 2008). These findings contrast with the Suzuki study where HFS induced-LTP was insensitive to mGluR blockade (Suzuki et al., 2001b). Additionally, post-pre stimulation plasticity was inhibited by NMDA receptor blockade, suggesting that HFS induced-LTP occurs by another mechanism. Further exploration into LTP/LTD of the excitatory inputs to ChIs represents an exciting field of study, as changes in synaptic strength here may contribute to motor skill learning and habit formation.

## Summary

Even though ChIs only make up 1–2% of all striatal cells, they send dense projections throughout the striatum. ACh can affect the output of the striatum directly or indirectly, and a wide variety of neurotransmitter systems can influence the activity of these cells. Given that ChIs are in a position to integrate synaptic inputs and modulate the output of the striatum, understanding the physiology of these cells will contribute to our knowledge of striatal function.

ChIs receive afferent inputs from a wide variety of sources which can arise either locally from within the striatum, or from brain regions as distant as the brainstem. Some of these neurotransmitter systems alter cellular excitability rapidly through their actions on ionotropic receptors, producing rapid electrical signals on a millisecond time scale. These changes in membrane properties affect the firing activity of the ChIs, and given the hundreds of thousands of synaptic contacts formed by each ChI, several other cell types are influenced by changes ACh release. The inherent membrane properties of ChIs allows them to be easily modified in either direction during neurotransmitter release: their depolarized resting membrane potential allows excitatory neurotransmitters to easily enhance action potential firing rate, and considering their tonic activity, inhibitory neurotransmitters will inhibit firing, reducing total cholinergic tone. Thus, because these cells are resting at some intermediate state of activity, they are sensitive to incoming afferents. In addition to the ionotropic receptors on these cells, ChIs express a wide variety of GPCRs. Activation of GPCRs can have a multitude of cellular effects, including the opening of ion channels, changes in plasticity or protein transcription (Altier, 2012; Rojas and Dingledine, 2013). The long time course of the signaling cascades downstream of GPCR activation could indicate that a temporary increase in neurotransmitter activity may lead to long-lasting modifications in ChI physiology that increases or decreases the cellular response to other incoming afferents. Given the sensitivity of these cells to various synaptic inputs, understanding ChI connectivity provides insight into the striatal network.

ACh has a wide variety of effects following release, either by directly activating receptors on postsynaptic cells or indirectly via the modulation of neurotransmitter release at terminals. Changes in striatal cholinergic tone will thus result in a complex series of downstream effects which ultimately may affect the striatal output neurons. Considering the multiple ACh-sensitive neurotransmitter systems that are involved in the striatal network, changes in receptor function or expression on any class of cells may result in a shift in the balance of these systems, potentially resulting in dysfunction. Understanding the nature of synaptic connectivity and the location of receptor expression therefor has a direct connection with human pathology. Using this knowledge in conjunction with an understanding of the changes that occur in disease, we can work toward the development of novel therapies that are aimed at counteracting neurotransmitter dysregulation.

Often times, we oversimplify the nature of the neurotransmitters released at a given terminal, neglecting the co-release of other neurotransmitters whose post-synaptic effects can differ from the primary neurotransmitter. Although there is evidence that co-release occurs, very few have looked in detail at the functional consequences of multiple transmitter release, or whether or not these neurotransmitters are released in sufficient amounts to contribute to cell physiology.

New genetic techniques can improve our understanding of striatal neurotransmission in both the normal and abnormal brain. Optogenetic manipulation of excitability in specific neuronal subtypes is providing important insights into connectivity throughout the nervous system. This robust technology has advantages over other methods of exogenous neural control and certainly provides a means to explore ChI efferent and afferent connections, as well as the nature of the neurotransmitter phenotypes that influence the excitability of these neurons. Extending these methods to *in vivo* analyses can help provide causal links between synaptic information and behavior.

CLARITY is a potentially groundbreaking new technique that allows high resolution visualization of subcellular structures such as individual synapses in the whole brain (Chung and Deisseroth, 2013). Because the preparation for CLARITY removes brain lipids, antibodies can easily permeate the entirety of the brain, permitting the resolution of the striatal connectome in total. This anatomical information will precisely elucidate the synaptic connectivity between striatal cell types.

Ultimately, ongoing efforts to improve our understanding of striatal ChIs will provide valuable insights into the physiology of this important brain area and help identify new pharmacotherapies for striatal disorders.

### Conflict of interest statement

The authors declare that the research was conducted in the absence of any commercial or financial relationships that could be construed as a potential conflict of interest.
